# Primary spinal glioblastoma: A case report and review of the literature

**DOI:** 10.3892/ol.2012.1076

**Published:** 2012-12-14

**Authors:** NUNO MORAIS, LINO MASCARENHAS, J.P. SOARES-FERNANDES, ANA SILVA, ZITA MAGALHÃES, J.A. MOREIRA DA COSTA

**Affiliations:** 1 Department of Neurosurgery, Hospital de Braga, Braga;; 2 Department of Neurosurgery, Centro Hospitalar Vila Nova de Gaia/Espinho, Vila Nova de Gaia;; 3 Departments of Neuroradiology, Hospital de Braga, Braga, Portugal; 4 Anatomic Pathology, Hospital de Braga, Braga, Portugal

**Keywords:** spinal cord tumours, intramedullary gliomas, spinal glioblastoma, metastases, radiotherapy, chemotherapy

## Abstract

Primary spinal glioblastoma (GBM) is a rare disease, with an aggressive course and a poor prognosis. We report a case of a 19-year-old male with a 4-week history of progressive weakness in both lower limbs, which progressed to paraparesis with a left predominance and difficulty in initiating urination over a week. Spine magnetic resonance imaging (MRI) showed an intramedullary expansile mass localised between T6 and T11. We performed a laminotomy and laminoplasty between T6 and T11 and the tumour was partially removed. Histopathological study was compatible with GBM. The patient was administered focal spine radiotherapy with chemotherapy with temozolamide. Serial MRI performed after the initial surgery demonstrated enlargement of the enhancing mass from T3 to T12 and subarachnoid metastatic deposits in C2 and C4, the pituitary stalk, inter-peduncular cistern, left superior cerebellar peduncle and hydrocephalus. We review the literature with regard to the disease and treatment options, and report the unique features of this case. Primary spinal GBM is an extremely rare entity with a poor prognosis and a short survival time. An aggressive management of the different complications as they arise and improvement of current modes of treatment and new treatment options are required to improve survival and ensure better quality of life.

## Introduction

Astrocytoma is the most common primary tumour of the central nervous system (1). Spinal neoplasms in the adult population are mostly extradural (55%) and intradural extra-medullary tumours (40%), whereas intramedullary tumours account for 5% of all spinal cord tumours, excluding metastatic lesions (2). Of these, ∼30% are tumours of low malignancy, including slow-growing astrocytomas and ependymomas. Spinal cord astrocytomas are rare, representing ∼1% of all primary central nervous system tumours and 6 to 8% of all spinal cord tumours (3). Few spinal cord astrocytomas are anaplastic in nature; most are slow-growing lesions. The ratio of low-grade to high-grade astrocytomas in the spinal cord is ∼3 to 1 (4,5). Glioblastomas (GBMs) represent ∼7.5% of all intramedullary gliomas and 1 to 3% of all spinal cord tumours (4,6). Moreover, GBM has a predilection of development at the cervical or cervicothoracic region in >60% of cases (1,7–9). Clinical presentation is associated with the region of spinal cord involved, irrespective of tumour type. Unlike their intracranial counterpart, intramedullary GBMs have received scant attention in the literature, with <200 cases reported. Even with aggressive management, these tumours are generally associated with a dismal outcome.

## Case report

A 19-year-old male was transferred to our institution in May 2010 with a 4-week history of progressive weakness in both lower limbs, which progressed to paraparesis with a left predominance and difficulty in initiating urination over a week. Examination revealed spastic paraparesis (right/left: grade 4+ and 3+, respectively) and hypoesthesia below the T10 sensory dermatome.

The study was approved by the Ethics Committee of Hospital de Braga, Braga, Portugal. Informed consent was obtained from the patient’s family.

**Figure 1:**
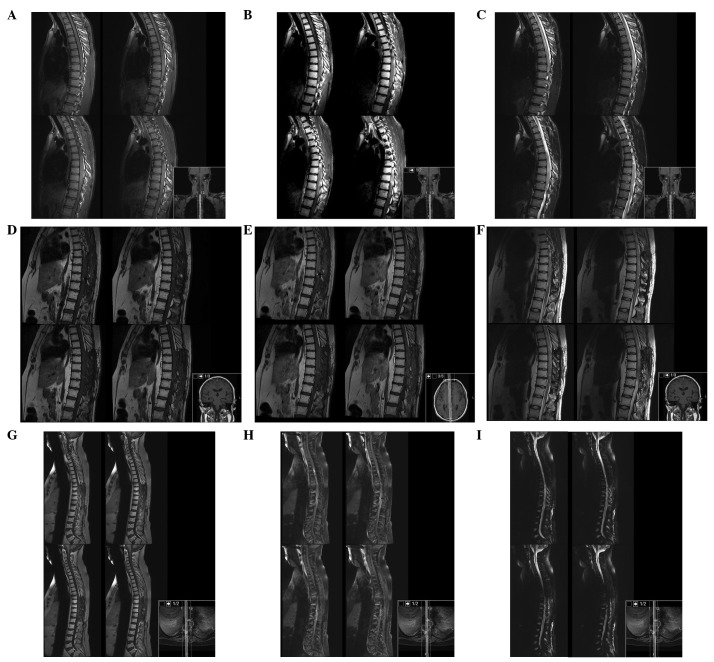
Spine MRI. (A) T1, (B) T1 with contrast and (C) T2-weighted dorsal spine sagittal view MRI at presentation, demonstrating the presence of the tumour between T1 and L1. (D) T1, (E) T1 with contrast and (F) T2-weighted dorsal spine sagittal view MRI 3 months after surgery. (G) T1, (H) T1 with contrast and (I) T2-weighted dorsal spine sagittal view MRI 17 months after surgery, demonstrating enlargement of the enhancing mass from T3 to T12 with subarachnoid metastatic deposit in C2 and C4. MRI, magnetic resonance imaging.

The patient underwent brain and spine magnetic resonance imaging (MRI). The brain and cervical spine were negative for masses and signal intensity alterations, whereas from T1 to L1 there was a marked spinal cord signal intensity and morphology alteration, with notable spinal cord expansion between T6 and T11 and contrast enhancement between T6 and T9 (Fig. 1).

We performed a laminotomy and laminoplasty between T6 and T11, and partial tumour removal under motor-evoked potential monitoring. We were unable to distinguish the tumour margin from the spinal cord and decided to partially remove the mass.

**Figure 2:**
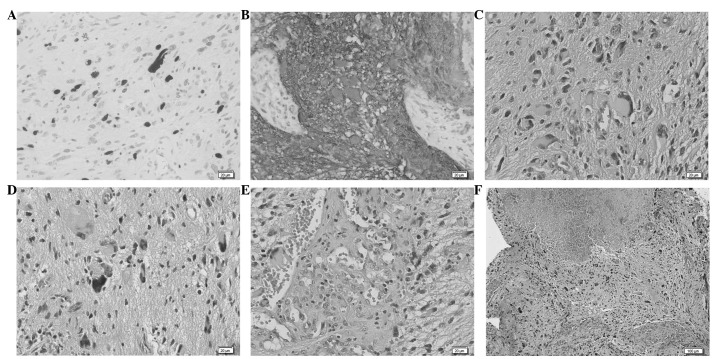
Histological findings of glioblastoma. (A) High MIB-1 /Ki-67 labelling index. (B) Marked immunoreactivity for GFAP. (C and D) Pleomorphic glial tumour cells with marked nuclear atypia. (E) Vascular proliferation was observed throughout the lesion. (F) Presence of extensive areas of necrosis. (A–E) Magnification, ×400. (F) Magnification, ×100. (C–F) Hematoxylin/eosin staining. GFAP, glial fibrillary acidic protein.

Histopathological study confirmed the diagnosis of GBM (Fig. 2), with histological findings of pleomorphism, atypical cells with high cellularity, vascular proliferation and necrosis. As demonstrated by immunohistochemistry, glial fibrillary acidic protein (GFAP) and S100 Protein were consistently expressed by tumour cells. The neoplasm also showed a high MIB1/Ki-67 labelling index.

Post-operatively the patient had transient neurological deterioration with worsening of paraparesis, but with intensive rehabilitation his condition returned to baseline. Thoracoabdominal CT scan for extra-neuronal metastases was negative. The patient was administered spinal radiotherapy between T1 and L1 (45 Gy in 28 fractions) with chemotherapy with temozolamide (completed 6 cycles).

**Figure 3:**
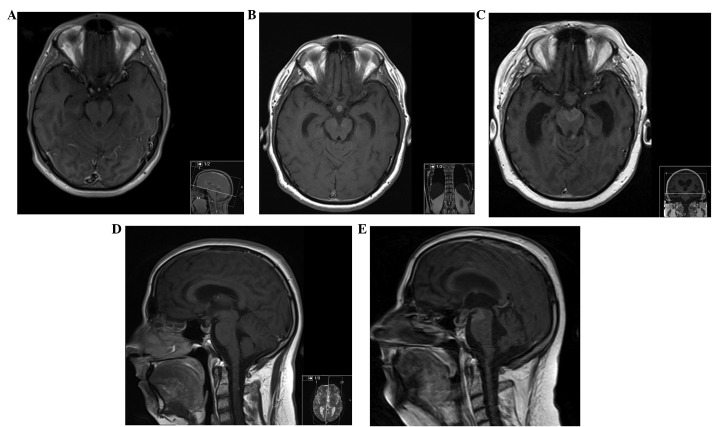
MRI of the brain. (A) Axial section, contrast-enhanced T1-weighted MRI of the brain, at presentation, demonstrating no lesions. (B and D) Axial and sagittal section, respectively, contrast-enhanced T1-weighted MRI of the brain, at 6 months after initial presentation, showing a metastatic deposit in the pituitary stalk. (C) Axial and (E) sagittal section contrast-enhanced T1-weighted MRI of the brain, at 17 months after initial presentation, demonstrating new metastatic deposits in the interpeduncular cistern and in the left superior cerebellar peduncle. MRI, magnetic resonance imaging.

Serial MRI at 3, 6 and 17 months was performed (Fig. 3). Six months after surgery the patient deteriorated, becoming completely paraplegic and losing bladder function. Cranial and spine MRI revealed enlargement of the residual tumour from T3 to T12 with cranial extension of oedema to the obex, and subarachnoid metastatic deposits in C2, C4 and in the pituitary stalk, with hydrocephalus. We proposed cranio-spinal irradiation, but as the patient was stable and without signs or symptoms of increased intracranial pressure (ICP) the patient and his family decided to withhold this treatment and wait.

Neuroaxis MRI performed 17 months after surgery revealed an enlargement of the enhancement mass from T3 to T12 with less perilesional oedema and a new metastatic deposit in the interpeduncular cistern and left superior cerebellar peduncle.

On the follow-up 20 months after surgery, the patient presented with a left third cranial nerve paralysis and bilateral mydriasis without signs of increased ICP or mental status change. CT scan showed larger ventricles and enlargement of the pituitary stalk and left superior cerebellar peduncle metastatic deposits. Given the disease progression and the Karnofsky score (40%), we did not consider further treatment and the patient succumbed to the disease 1 month later.

## Discussion

Intramedullary GBM is a rare disease entity. It develops primarily from the spinal cord or as a secondary metastasis from the brain, which covers up to 25% of the total occurrences. Intramedullary GBM has a predilection to develop from the cervical region in primary cases, and has a tendency to develop at a young age (<30 years old). Despite the best treatment (surgery and adjuvant therapy), the estimated survival barely exceeds 6 to 16 months (1,8–11).

Certain patients with malignant spinal cord astrocytomas develop hydrocephalus (1,7), which is thought to be due to increased protein concentration in the cerebrospinal fluid (CSF), occlusion of the CSF channel in the subarachnoid space at the skull base and brain surface, arachnoiditis and bleeding of spinal cord tumours (6,7).

Seeding of an intracranial GBM along the spine occurs in 25% of cases, but the reverse process is extremely uncommon (1,3). Patients with malignant spinal cord astrocytomas may develop disseminated disease, mostly via the leptomeningeal route (1,6); however, no definite evidence has been elucidated. Sites of intracranial metastases include the subarachnoid space, ventricles, cerebellum, hypothalamus, brain stem, thalamus and septum pellucidum. Surgical manipulation of GBM has not been shown to increase the tumour seeding into the CSF (3). Continuous spread to contiguous regions is rare, with the most common sites metastases being extra-neuronal, including the lungs, lymph nodes, bone, liver and pleura (12). In our case, metastases were observed in the subarachnoid space in C2 and C4, pituitary stalk, interpeduncular cistern and left superior cerebellar peduncle.

All current therapeutic measures have produced disappointing results and few data concerning their real value are available, with survival times between 6 and 16 months with a mean survival period of 12 months after diagnosis (1,4,7–10) Radical surgery is suggested for confirmation of the diagnosis and for cytoreduction of the tumour as an adjunct to radiotherapy and chemotherapy.

Most authors suggest focal spine radiotherapy and chemo-therapy with temozolamide, while others recommend a more aggressive approach with whole-brain irradiation in addition to focal spine irradiation, even if there is no evidence of intracranial dissemination. Others suggest intrathecal administration of interferon-β via an Ommaya reservoir in conjunction with cranio-spinal irradiation (3,7).

MRI is considered the gold standard imaging modality to diagnose intramedullary tumours (13,14), and gadolinium-enhanced MRI of the entire neuroaxis is advocated to rule out metastasis, evaluate treatment efficacy and detect relapse (15).

To the best of our knowledge, only 16 cases of spinal GBM involving the conus medullaris have been previously reported (1,4,13–19,20–24), making this case the first with spinal and intracranial metastasis with hydrocephalus and the third most longest survival (21 months).

In conclusion, primary spinal GBM is an extremely rare entity. Despite aggressive treatment with radical surgery, radiotherapy and chemotherapy, this disease progresses rapidly with a poor prognosis and a short survival time. We advocate an aggressive management of the different complications as they arise (progression, metastasis, hydrocephalus) to extend the patient’s survival as long as possible with the best quality of life. Improvement of current modes of treatment and new treatment options (chemotherapy protocols, gene therapy) are required to improve survival and ensure better quality of life.

## Abbreviations:

GBM: glioblastoma
CSF: cerebrospinal fluid
MRI: magnetic resonance imaging
ICP: intracranial pressure

## References

[1] Cohen AR, Wisoff JH, Allen JC, Epstein F (1989). Malignant astrocytomas of the spinal cord. J Neurosurg.

[2] Balériaux DL (1999). Spinal cord tumors. Eur Radiol.

[3] Johnson D, Schwarz S (1987). Intracranial metastases from malignant spinal-cord astrocytoma. Case report. J Neurosurg.

[4] Medhkour A, Chan M (2005). Extremely rare glioblastoma multiforme of the conus medullaris with holocord and brain stem metastases, leading to cranial nerve deficit and respiratory failure: A case report and review of the literature. Surg Neurol.

[5] Stein BM (1979). Surgery of intramedullary spinal cord tumors. Clin Neurosurg.

[6] Ciappetta P, Salvati M, Capoccia G, Artico M, Raco A, Fortuna A (1991). Spinal glioblastoma: report of seven cases and review of the literature. Neurosurgery.

[7] Asano N, Kitamura K, Seo Y (1990). Spinal cord glioblastoma multiforme with intracranial dissemination - case report. Neurol Med Chir (Tokyo).

[8] Grisold W, Pernetzky G, Jellinger K (1981). Giant-cell glioblastoma of the thoracic cord. Acta Neurochir (Wien).

[9] Guidetti B, Mercuri S, Vagnozzi R (1981). Long-term results of the surgical treatment of 129 intramedullary spinal gliomas. J Neurosurg.

[10] Alvisi C, Cerisoli M, Giulioni M (1984). Intramedullary spinal gliomas: long-term results of surgical treatments. Acta Neurochir (Wien).

[11] Kopelson G, Linggood RM (1982). Intramedullary spinal cord astrocytoma versus glioblastoma: the prognostic importance of histological grade. Cancer.

[12] Russell DS, Rubinstein LJ (1998). Glioblastoma. Pathology of Tumours of the Nervous System.

[13] Bonde V, Balasubramaniam S, Goel A (2008). Glioblastoma multiforme of the conus medullaris with holocordal spread. J Clin Neurosci.

[14] Stecco A, Quirico C, Giampietro A, Sessa G, Boldorini R, Carriero A (2005). Glioblastoma multiforme of the conus medullaris in a child: description of a case and literature review. AJNR Am J Neuroradiol.

[15] Mori K, Imai S, Shimizu J, Taga T, Ishida M, Matsusue Y (2012). Spinal glioblastoma multiforme of the conus medullaris with holocordal and intracranial spread in a child: a case report and review of the literature. Spine J.

[16] Andrews AA, Enriques L, Renaudin J, Tomiyasu U (1978). Spinal intramedullary glioblastoma with intracranial seeding. Report of a case. Arch Neurol.

[17] Eden KC (1938). Dissemination of a glioma of the spinal cord in the leptomeninges. Brain.

[18] Kawanishi M, Kuroiwa T, Nagasawa S, Ohta T, Oketa M, Onomura T (1993). A case of spinal glioblastoma with intracranial dissemination. No Shinkei Geka.

[19] O’Connell JE (1946). The subarachnoid dissemination of spinal tumours. J Neurol Neurosurg Psychiatry.

[20] Santi M, Mena H, Wong K, Koeller K, Olsen C, Rushing EJ (2003). Spinal cord malignant astrocytomas. Clinicopathologic features in 36 cases. Cancer.

[21] Scarrow AM, Rajendran P, Welch WC (2000). Glioblastoma multiforme of the conus medullaris. Clin Neurol Neurosurg.

[22] Shirato H, Kamada T, Hida K (1995). The role of radiotherapy in the management of spinal cord glioma. J Radiat Oncol Biol Phys.

[23] Strik HM, Effenberger O, Schäfer O, Risch U, Wickboldt J, Meyermann R (2000). A case of spinal glioblastoma multiforme: immunohistochemical study and review of the literature. J Neurooncol.

[24] Tashiro K, Tachibana S, Tsura M (1976). Clinicopathological studies of spinal cord neoplasm with disseminating intracranial metastasis possibly producing akinetics mutism. No To Shinkei.

